# The clinical significance of MMP-1 expression in oesophageal carcinoma

**DOI:** 10.1054/bjoc.2000.1568

**Published:** 2001-01

**Authors:** K Yamashita, M Mori, A Kataoka, H Inoue, K Sugimachi

**Affiliations:** Department of Surgery, Medical Institute of Bioregulation, Kyushu University, Beppu, Japan; Department of Surgery II, Faculty of Medicine, Kyushu University, Fukuoka, Japan

**Keywords:** MMP-1, mRNA, oesophageal carcinoma, invasion, prognosis

## Abstract

Matrix metalloproteinase-1 (MMP-1) is involved in the degradation of interstitial collagen and thus thought to play a role in invasion of carcinoma. We investigated 51 oesophageal carcinoma patients to clarify the significance of MMP-1. MMP-1 mRNA was demonstrated to be expressed exclusively in almost all carcinoma tissue specimens (T) (94.1%) by reverse transcription-polymerase chain reaction, but not found in normal mucosal tissue specimens (N). The mean T/N ratio of MMP-1 was 42.5 and cases with T/N ≥ 10 had a higher incidence of cases involving muscularis propria than those with T/N < 10 which included all the cases involving the submucosa (*P*< 0.05). MMP-1 mRNA was significantly associated with both 40 kD (putative active MMP-1) and 50 kD (putative latent MMP-1) gelatinolytic bands (*n* = 17). These findings indicated that MMP-1 mRNA reflected the net function of MMP-1 and suggested MMP-1 to be involved in carcinoma invasive process. On the other hand, MMP-1 mRNA was inversely correlated with the patient prognosis (*P*< 0.01). These results indicated that MMP-1 might therefore play a crucial role in local invasion, but not in systemic dissemination. As a result, MMP-1 might be a novel prognostic factor independent from those previously reported in oesophageal carcinoma. © 2001 Cancer Research Campaign http://www.bjcancer.com


British Journal of Cancer (2001) **84**(2), 276–282
© 2001 Cancer Research Campaign
doi: 10.1054/ bjoc.2000.1568, available online at http://www.idealibrary.com on IDEAL®

http://www.bjcancer.com

### The clinical significance of MMP-1 expression in oesophageal carcinoma

**K Yamashita,[1] M Mori,[1] A Kataoka,[1] H Inoue,[1] and K Sugimachi[2]**

[1]Department of Surgery, Medical Institute of Bioregulation, Kyushu University, Beppu, Japan; [2]Department of Surgery II, Faculty of Medicine, Kyushu University, Fukuoka, Japan

**Summary** Matrix metalloproteinase-1 (MMP-1) is involved in the degradation of interstitial collagen and thus thought to play a role in invasion of carcinoma. We investigated 51 oesophageal carcinoma patients to clarify the significance of MMP-1. MMP-1 mRNA was demonstrated to be expressed exclusively in almost all carcinoma tissue specimens (T) (94.1%) by reverse transcription-polymerase chain reaction, but not found in normal mucosal tissue specimens (N). The mean T/N ratio of MMP-1 was 42.5 and cases with T/N ≥ 10 had a higher incidence of cases involving muscularis propria than those with T/N < 10 which included all the cases involving the submucosa ($P < 0.05$). MMP-1 mRNA was significantly associated with both 40 kD (putative active MMP-1) and 50 kD (putative latent MMP-1) gelatinolytic bands ($n = 17$). These findings indicated that MMP-1 mRNA reflected the net function of MMP-1 and suggested MMP-1 to be involved in carcinoma invasive process. On the other hand, MMP-1 mRNA was inversely correlated with the patient prognosis ($P < 0.01$). These results indicated that MMP-1 might therefore play a crucial role in local invasion, but not in systemic dissemination. As a result, MMP-1 might be a novel prognostic factor independent from those previously reported in oesophageal carcinoma. © 2001 Cancer Research Campaign http://www.bjcancer.com

**Keyword**: MMP-1; mRNA; oesophageal carcinoma; invasion; prognosis

Matrix metalloproteinases (MMPs) play a crucial role in carcinoma invasion (Ossowski, 1992), metastasis (Liotta et al, 1991; Kim et al, 1998), angiogenesis (Brooks et al, 1998), and tumorigenicity (Wilson et al, 1997; Masson et al, 1998). MMP-1 is called interstitial collagenase because of the efficient degradation of interstitial collagen mainly composed of type I, II and III collagen (Woessner, 1991). The spread of carcinoma cells was demonstrated to correlate with the lysis of type I collagen, which is the principal component of connective tissues at most body sites, in colorectal carcinoma (van der Stappen et al, 1990) and head and neck carcinoma (Burman and Carter, 1985). Ossowski (1992) also revealed that invasion of connective tissue by carcinoma cells particularly required MMP-1. MMP-1 thus appears to be involved in carcinoma invasion. On the other hand, type IV collagenolytic activity, which is known to be associated with the disruption of basement membrane, plays a very important role in the distant metastatic potential of carcinoma cells (Liotta et al, 1980). MMP-1 could, however, hardly degrade type IV collagen, which indicates that MMP-1 is not closely involved in penetration of vessels. Therefore MMP-1 may not be directly associated with distant metastatic potential. Moreover, MMP-2 and MMP-9, which are called type IV collagenase due to the efficient degradation of type IV collagen, is less able to degrade interstitial collagen (Woessner, 1991). These findings suggest that carcinoma invasion and metastasis are somewhat independent processes.

Murray et al claimed that MMP-1 immunolocalized in carcinoma cells could be associated with a poor prognosis in colorectal (Murray et al, 1996) and oesophageal carcinoma (Murray et al, 1998b). However, their investigation of oesophageal carcinoma included 19 cases of squamous cell carcinoma and only 3 cases (16%) which expressed MMP-1. In Japan, oesophageal carcinoma consists largely of squamous cell carcinoma and Shima et al (Shima et al, 1992) demonstrated that oesophageal squamous cell carcinoma showed a positive correlation with the depth of invasion, lymph node metastasis, and vessel permeation regarding MMP-2 and MMP-3 expression, but not MMP-1. However, the presence of protein does not necessarily reflect the net function, and recent studies have shown mRNA (e.g. MT1-MMP mRNA) to possibly reflect the net function (activated MMP-2) in clinical carcinoma tissue (Mori et al, 1997; Yoshizaki et al, 1997). We thus investigated MMP-1 mRNA expression of oesophageal carcinoma, which was mainly composed of squamous cell carcinoma, in order to clarify the significance of MMP-1.

*Received 23 March 2000*
*Revised 25 August 2000*
*Accepted 25 September 2000*

*Correspondence to:* M Mori

### MATERIALS AND METHODS

### Patients and sample collection

51 oesophageal carcinomas and their paired normal mucosa from patients who underwent surgery at the Medical Institute of Bioregulation Hospital, Kyushu University and the Saitama Cancer Center had MMP-1 mRNA levels assessed. All patients had undergone resection of the primary carcinoma, and none had received chemotherapy or radiotherapy. The patients included 45 males and 6 females. The tumour was located either in the upper oesophagus ($n = 3$), the middle oesophagus ($n = 36$), or the lower oesophagus ($n = 12$). Twelve tumours were well differentiated, 25 were moderately differentiated, and 12 were poorly differentiated squamous cell carcinomas. Other types of carcinoma included one basal cell carcinoma and one adenocarcinoma. The depth of invasion of the tumour was as follows, 5 involved the submucosa, 7 involved the muscularis propria, and 39 involved the adventitia or more. The cases with lymph node metastases were classified into two groups: the non-metastatic group (*n* = 8) and metastatic group (*n* = 43). Carcinoma specimens were obtained from the tumour edge, avoiding a necrotic centre and corresponding distant normal mucosa specimens were also obtained at least 5 cm away from the tumour edge by sharply dissecting the mucosa off the muscularis propria. All specimens were quick-frozen in liquid nitrogen and stored at −80˚C until processing.

When obtaining the tumour tissue from the surgical specimens, we have carefully chosen the area where abundant tumour cells seemed to be contained. The cut surface of the tumour centre indicate the whitish area where usually contain a lot of tumour cells. Moreover we reviewed the specimens histologically, and almost all specimens (47/51) were ascertained that more than 80% area contained tumour cells and the remaining 4 showed that tumour cells occupied more than 70%. Thus we think the used specimens were good for the molecular analysis.

### RNA extraction, reverse transcription-polymerase chain reaction (RT-PCR) and Northern blot hybridization

### *Total RNA isolation*

Frozen-tissue specimens were homogenized in guanidium thiocyanate and total RNAs were obtained by ultracentrifugation through a cesium chloride cushion as described previously (Mori et al, 1993; Yamashita et al, 2000).

### *cDNA preparation and RT-PCR*

Eight µg of each total RNA was reverse-transcribed with M-MLV reverse transcriptase (GIBCO BRL Inc., Rockville, MD), and one-hundredths of the products were amplified by PCR according to the following manner as previously reported (Mori et al, 1997). The oligonucleotide primer pairs for MMP-1 were purchased from Amersham Pharmacia Biotech UK Incorporation (Little Chalfont, UK) (sense MMP-1, 5'-TCGCTGGGAGCAAACACATC-3'; antisense MMP-1, 5'-TTCATGAGCCGCAACACGAT-3'). It was carried out in a 30 µl volume containing cDNA template, 10 pmol each of oligodeoxynucleotide primer, 200 mM each of deoxynucleotide triphosphate, 200 mM deoxycytidine triphosphate labelled with $\alpha$-$^{32}$P, PCR buffer with 1.5 mM magnesium chloride (Perkin Elmer Cetus, Norwalk, CT), and 1.5 U of Taq polymerase (Perkin Elmer Cetus, Norwalk, CT). The samples were overlaid with mineral oil and processed through 24 cycles consisting of 1 min at 95˚C (denaturation), 1 min at 56˚C (annealing), 1 min at 72˚C (elongation). The same aliquots of the amplified DNA by PCR reaction were mixed with formamide gel loading buffer and then were electrophoresed on 5% polyacryl amide gels. The gels were dried, and exposed to an imaging plate. The samples were then quantitated using a Bio-Image analyzer BAS 1000 (Fuji Photo Inc., Tokyo, Japan). The mRNA expression in tumour (T) and normal (N) tissue in each pair was estimated on the basis of the counts obtained. The tumour/normal ratio (T/N ratio) of MMP-1 was calculated with or without correcting for the GAPDH expression in the following manner: T/Nraw = MMP-1 (T) / MMP-1 (N), T/Nc (T/N in the following text) = (MMP-1 (T) / GAPDH (T)) / (MMP-1 (N) / GAPDH (N)). PCR products were ligated into pCR 2.1 vector (In Vitrogen Inc. Carlsbad, CA) and sequencing

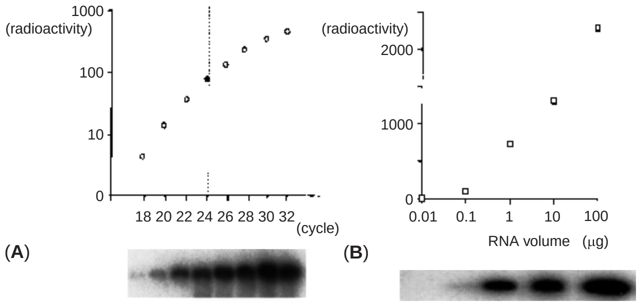

**Figure 1** (**A**) The radioactivity of PCR products and the number of PCR cycles. The radioactivity increased linearly between 18 and 32 PCR cycles for MMP-1. (**B**) The radioactivity of PCR products and serial dilutions of RNA samples. The radioactivity of PCR products correlated with the RNA content confirmed MMP-1 using DNA sequencing kit-Dye terminator cycle sequencing ready reaction (Perkin Elmer Applied Biosystems Inc., Foster, CA). To validate the quantitative analysis for MMP-1 expression using our RT-PCR method, the radioactivity for MMP-1 were examined based on varying numbers of PCR cycles and serial dilutions of RNA samples. Figure 1 showed the radioactivity values to increase linearly at 24 cycles and also showed a correlation with the RNA contents. These findings thus indicated a quantitative assessment in this assay for MMP-1. The appropriate cycle for glyceraldehyde 3-phosphate dehydrogenase (GAPDH) was determined according to the previously described method (Mori et al, 1997).

### *Northern blot hybridization*

Equal amounts (15 µg) of total RNA were loaded and analysed as previously described (Mimori et al, 1996). The fold-increase of MMP-1 in each tumour tissue specimen relative to its corresponding adjacent normal tissue specimen was calculated after correcting for the GAPDH expression. MMP-1 probe was kindly conferred by Professor Barry L. Marmer, Division of Dermatology, School of Medicine, Washington University.

### Gelatin zymography

SDS-PAGE and zymography using gelatin containing gel for detecting gelatinolytic activities were performed in 17 cases following the procedures reported previously (Mori et al, 1997). Briefly, each specimen was homogenized in a sample buffer containing 10 mM Tris/HCl, pH 6.8, 20% glycerin, 2% SDS, and 0.1% bromophenol blue. The samples were separated by electrophoresis on 10% polyacrylamide gel containing 0.1% SDS and 1 mg ml$^{-1}$ gelatin as a substrate. The gels were then washed in the renaturation buffer (50 mM Tris/HCl, pH 7.5, 0.1 M NaCl) containing 2.5% Triton X-100 for 90 minutes. Thereafter the gels were incubated for 18 hours at 37˚C in a reaction buffer (50 mM Tris/HCl, pH 7.5, 10 mM $CaCl_2$) and stained with 0.1% Coomassie Brilliant blue R250.

### Western blot analysis

Total protein was extracted from clinical samples (*n* = 10) by RIPA buffer. This same aliquots (50 µg) of total protein were applied to 10% acrylamide gradient gels. Following electrophoresis, samples were electroblotted onto a polyvinylidene difluoride membrane

*© 2001 Cancer Research Campaign*

(Immobilin, Millipore Inc., Bedford, MA) at 0.5 A for 1.5 hours at 4˚C. Both MMP-1 and MMP-2 were detected by the use of mouse monoclonal primary antibodies (F-67 and F-68, respectively, Fuji Pharmacochemical Inc., Toyama, Japan) used in an a dilution of 1:1000. The blots were developed using anti mouse immunoglobulin, horseradish peroxidase liked whole antibody (Promega Inc., Madison, WI). Signals were detected using Supersignal (Pierce Inc., Reckford, IL). The prestained high molecular-weight markers were run on gels (Amersham Life Science Inc., Little Chalfont, England).

### Immunohistochemistry

To identify the localization of MMP-1 protein in the oesophageal carcinoma, an immunohistochemical analysis was performed in 17 oesophageal carcinoma cases by using a previously described method (Mori et al, 1997). Six normal oesophageal mucosas were investigated in the same manner. Briefly, 5 micron thick sections were cut from the formalin fixed, paraffin-embedded block. MMP-1 was detected by the use of mouse monoclonal primary antibody to MMP-1 (F-67, Fuji Pharmacochemical Inc., Toyama, Japan) in a dilution of 1:100. Immunostaining was done by the avidin-biotin-peroxidase method.

### Quantitation and statistical analysis

Associations between the variables were tested by Student's *t* test or Fisher's exact probability test. Survival analysis was done by Cox-Mantel test.

### RESULTS

### MMP-1 expression at mRNA level in oesophageal carcinoma

Our present analysis of oesophageal carcinoma showed that MMP-1 was almost exclusively expressed in the carcinoma tissue specimens. RT-PCR was very sensitive and 48 carcinoma tissues (94.1%) expressed MMP-1 mRNA in 51 patients and almost non-expression in normal oesophageal tissues (Fig. 2). In RT-PCR, the value of MMP-1 T/N ratio corrected for GAPDH was mean 42.5 (0.3–277.4). With respect to T/N ratio of GAPDH mRNA expression, the average was 1.3 varying from 0.5 to 2.3 in RT-PCR. To confirm the accuracy of quantitation, we performed a Northern blot analysis in as many specimens as we could ($n$ = 45) and in 21 (47%) of the 45 cases, the expression of MMP-1 mRNA was recognized in the tumour tissue, while almost no expression for MMP-1 was recognized in the normal oesophageal tissue. T/N ratio of MMP-1 mRNA after correction of GAPDH ranged from 0.4 to 42.6 (mean 11.5) in a Northern analysis and almost all cases were confirmed to be equal between tumour and normal tissues when evaluated by the staining of Etidium Bromide as shown in Figure 2. In the range to be detected by a Northern blot analysis, the signal intensities by RT-PCR were closely related to the corresponding signals of a Northern blot analysis (R = 0.89; $P < 0.001$; data not shown) (Fig. 2).

### MMP-1 mRNA and clinicopathological factors

In a clinicopathological analysis, we established a cut off value for the MMP-1 T/N ratio in RT-PCR analysis to determine the

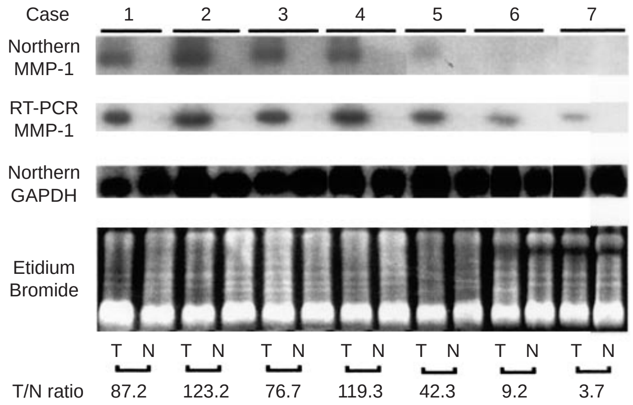

**Figure 2** A Northern blot and RT-PCR analysis of MMP-1 mRNA expression in oesophageal carcinoma tissues (T) and normal mucosal tissues (N). In both analyses, MMP-1 mRNA is expressed exclusively in T, but not in N. The same membranes of the top panel were stripped and rehybridized with a probe to GAPDH as an internal control. RNA was also visualized by the staining of Etidium Bromide and confirmed that equal volumes were loaded in each case. At the bottom panel, T/N ratio of MMP-1 mRNA after normalizing GAPDH in an RT-PCR is represented

**Table 1** Clinicopathological characteristics as to MMP-1 mRNA expression in oesophageal carcinoma

| | **MMP-1 T/N** | | |
|---|---|---|---|
| | **< 10 (*n* = 23)** | **$\geqq$ 10 (*n* = 28)** | ***P* value** |
| Age (y) | 64.8 ± 8.4 | 61.1 ± 10.0 | N.S. |
| Sex | | | |
| Male | 20 | 25 | |
| Female | 3 | 3 | N.S. |
| Histological differentiation[a] | | | |
| Well | 5 | 7 | |
| Moderately | 10 | 15 | |
| Poorly | 7 | 5 | |
| Others | 1 | 1 | N.S. |
| Location | | | |
| Upper | 3 | 0 | |
| Middle | 14 | 22 | |
| Lower | 6 | 6 | N.S. |
| Depth of invasion | | | |
| Submucosa | 5 | 0 | |
| Muscularis propria | 2 | 5 | ($P$ = 0.027) |
| Adventitia | 16 | 23 | $P < 0.05$ |
| Vascular permeation | | | |
| Absent | 5 | 7 | |
| Present | 18 | 21 | N.S. |
| Lymphatic permeation | | | |
| Absent | 2 | 3 | |
| Present | 21 | 25 | N.S. |
| Lymph node metastasis | | | |
| Absent | 3 | 5 | |
| Present | 20 | 23 | N.S. |
| Stage[b] | | | |
| 1, 2 | 5 | 3 | |
| 3, 4 | 18 | 25 | N.S. |

N.S. not significant. [a]This analysis included 49 squamous cell carcinoma. Others included one basal cell carcinoma and one adenocarcinoma. [b]According to the guidelines for clinical and pathological studies on carcinoma of the oesophagus. (Japanese Society for Esophageal Diseases, 1976).

© 2001 Cancer Research Campaign

**Table 2** *P* values and hazard ratios according to univariate Cox's analysis based on increasing cut-off values of MMP-1 in advanced oesophageal carcinoma

| Cut-off line | MMP-1 high (*n*) | MMP-1 low (*n*) | *P* value | Hazard ratio |
|---|---|---|---|---|
| 1 | 40 | 6 | 0 | 3.95 |
| 5 | 33 | 13 | 0.0018 | 3.13 |
| 10 | 28 | 18 | 0.0046 | 2.84 |
| 15 | 25 | 21 | 0.029 | 2.18 |
| 20 | 23 | 23 | 0.067 | 1.83 |

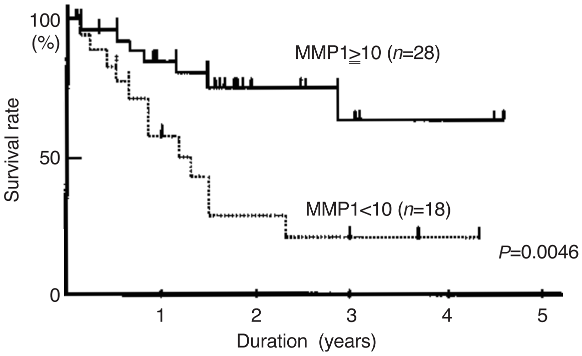

**Figure 3** Kaplan-Meier overall survival curves in oesophageal carcinomas excluding the cases involving the submucosa between high (T/N fold-increase ≥ 10) and low (T/N fold-increase < 10) MMP-1 mRNA expression by RT-PCR. The group with low MMP-1 expression showed a poorer prognosis than that with high MMP-1 expression. The Cox's analysis comparing the 2 groups revealed a significant difference (*P* = 0.0046)

malignant potential in each case. If a value of 10 was selected as a cut off line, then 23 cases with T/N < 10 had 5 patients involving the submucosa, while 28 cases with T/N ≥ 10 had no such patients (*P* = 0.027) (Table 1). Moreover, RT-PCR showed no expression in only 3 carcinoma tissue specimens, 2 of which involved submucosa (*P* = 0.023). Namely the group with a high MMP-1 expression showed a deeper invasion than that with a low MMP-1 expression. On the other hand, there were no significant differences regarding age, sex, histologic differentiation, lymph node metastasis, lymphatic or vascular permeation, or stage between these two groups by RT-PCR (Table 1).

We also established the various cut off values of the T/N ratio to determine the malignant potentiality-contribution of prognosis in advanced oesophageal carcinoma (Table 2). We selected as a cut-off line is 10 as described, where the number balance between high group (*n* = 18) and low group (*n* = 28) seemed appropriate. Therefore, T/N ratio of 10 was chosen in the following context. In our cases, no early stage carcinoma cases involving submucosa died despite T/N < 10 within the observed terms probably due to the early stage and small number of cases (*n* = 5). We therefore restricted our prognostic analysis to advanced cases (*n* = 46) involving the muscularis propria or more. The patients with T/N < 10 of MMP-1 showed a significantly poorer prognosis than those with T/N ≥ 10 of MMP-1, especially in advanced oesophageal carcinoma (*P* = 0.0046) (Fig. 3). We also investigated the T/N ratio of MMP-1 mRNA expression without correction by GAPDH in RT-PCR. MMP-1 mRNA T/N ratio after GAPDH correction caused only minor alteration of the order in each T/N ratio. In both analyses with or without correcting for GAPDH, the groups with high MMP-1 and low MMP-1 did not change when selecting a T/N ratio of 10 and therefore the results as shown in Figure 3 or Table 1 were not affected by this correction.

In contrast, 17 of 23 cases with T/N < 10 underwent a Northern blot analysis but MMP-1 mRNA was hardly detectable (T/N ratio ≤ 1) in all the cases. In contrast, all 28 cases with T/N ≥ 10 underwent a Northern blot analysis and 21 in all 28 cases were detectable (T/N ratio > 1). An analysis by a Northern blot hybridization also revealed a significant correlation with the depth of invasion (*P* = 0.03), but no other significant factors were observed (inverse tendency (*P* = 0.18) as to prognosis) (data not shown).

### Gelatinolytic band at 40 and 50 kD and MMP-1 mRNA association

Tumour tissue specimens and normal mucosa specimens of 17 oesophageal carcinoma cases were examined in gelatin zymography. All tumour samples showed a band at 72 kD which is a latent MMP-2 and 15 out of 17 showed a band at 66 or 64 kD which is an active MMP-2. The putative latent and active forms of MMP-1 migrated at 50 and 40 kD, respectively. 50 kD gelatinolytic band was demonstrated to be latent MMP-1 by immunoprecipitation with MMP-1 antibodies (Ossowski, 1992; Kim et al, 1998). All cases were evaluated at mRNA level of MMP-1 by both RT-PCR and Northern blot hybridization. Representative cases are shown in Figure 4A. Gelatin zymography demonstrated the latent form (50 kD) of MMP-1 to be present in 10 tumour (56%) and no normal tissue specimens were examined. On the other hand, the active form (40 kD) was presented in 9 (53%) tumour tissue samples and non-normal samples. Both 50 kD and 40 kD bands were closely associated with each other (Figure 4A). The 50 kD and 40 kD bands were also significantly associated with MMP-1 mRNA expression according to the Northern blot analysis (data not shown). Although 6 cases with MMP-1 containing tumours have very high MMP-2 activities, however, 3 cases with MMP-1 containing tumours have low MMP-2 activities as case 5 and 6 in Figure 4A, which is unlikely to suggest MMP-2 to be required for MMP-1 activation.

### Western blot analysis

Figure 4B showed representative bands of MMP-1 and MMP-2 corresponding to zymography in Figure 4A. In oesophageal carcinoma tissues, MMP-1 showed 50 kD (4/10) and 40 kD (3/10) bands and MMP-2 showed 72 (10/10), 66 (5/10), 64 (4/10), and 43 kD (1/10) bands. On the other hand, in normal oesophageal mucosal tissues, MMP-1 showed 50 kD (2/10) and 40 kD (0/10) bands and MMP-2 showed 72 (10/10), 66 (4/10), 64 (3/10), and 43 kD (0/10) bands. In the present analysis, there recognized no band at 50 kD using MMP-2 antibody.

### MMP-1 protein localization by immunohistochemistry

Six normal oesophageal mucosa samples showed weak immunostaining (Fig. 5A). Oesophageal carcinoma cells showed a varying degree of immunostaining for MMP-1 (11/17: 65%) (Fig. 5C),

© 2001 Cancer Research Campaign 

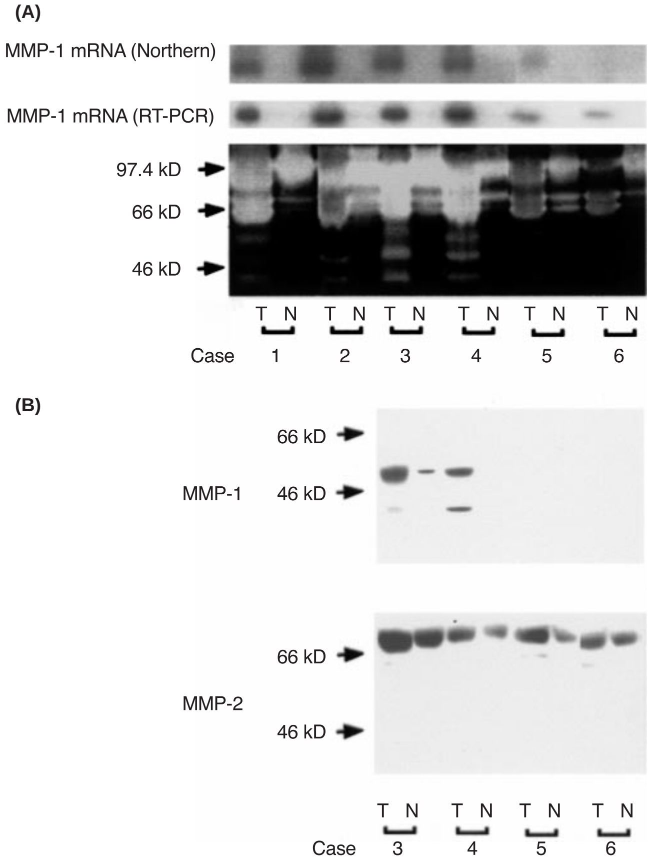

**Figure 4** (**A**) An analysis by gelatin zymography compared with MMP-1 mRNA expression in oesophageal carcinoma. The bands at 50 kD and 40 kD were recognized and significantly associated with each other. The normal mucosa revealed almost no these bands. Both bands were also associated with MMP-1 mRNA. (**B**) Western blot analysis demonstrated bands at 50 and 40 kD for MMP-1, and those at 72 and 64 kD for MMP-2 in oesophageal carcinoma and normal tissues. MMP-1 and MMP-2 protein are more abundant in oesophageal carcinoma tissues as compared with its corresponding normal mucosal tissues. As for MMP-2, the intensity of the bands at 72 kD was strong, while the bands at 64 kD showed very weak. These cases were identical ones shown in Figure 4A and might account for the results of gelatin zymography while some (4/17: 33%) for stromal cells (Fig. 5D). Either tumour or stromal immunostaining for MMP-1 was recognized in 13 (76%) of the 17 cases. Three in 17 showed marked eosinophil infiltration while the eosinophil showed an intense degree of staining for MMP-1 (Fig. 5E, F).

### DISCUSSION

Murray et al demonstrated that MMP-1 protein immunolocalized in carcinoma cells correlated with a poor prognosis in colorectal (Murray et al, 1996) and oesophageal carcinoma (Murray et al, 1998b), but not in gastric carcinoma (Murray et al, 1998a). However, in oesophageal carcinoma, the significance was minute regarding the prognosis and this report included 19 squamous cell carcinomas which showed only 3 cases (16%) of positive immunostaining of MMP-1 (Murray et al, 1998b). On the other hand, Shima et al also revealed MMP-1 protein expression in 21% of oesophageal squamous cell carcinoma but did not show any significant correlation with such malignant characteristics, while MMP-2 and MMP-3 showed a significant correlation with those characteristics (Shima et al, 1992). In contrast, in situ expression rate of MMP-1 mRNA was demonstrated to be high (70–100%) in various carcinoma tissues, which was concorded with our RT-PCR result, and mainly in stromal cells such as fibroblasts (Otani et al, 1994) and eosinophils (Gray et al, 1993; Ono et al, 1997). Discrepancies between mRNA and protein regarding the rate of expression and in situ localization remain controversial points of MMP-1 and so MMP-1 mRNA and protein would better be separately discussed (Otani et al, 1994; Murray et al, 1996).

We therefore investigated MMP-1 mRNA expression in oesophageal carcinoma and confirmed it to be expressed exclusively in almost all carcinoma tissues, but not in the normal mucosa (Fig. 2). This suggested that MMP-1 might play a significant role in carcinoma tissue. Three carcinoma tissues showing no expression of MMP-1 by RT-PCR included two cases involving submucosa ($P < 0.05$). Moreover, 28 cases with T/N ≥ 10 did not include any cases involving the submucosa, while 23 cases with T/N <10 included all 5 such cases ($P < 0.05$) (Table 1). These findings indicated that MMP-1 mRNA was correlated with the depth of invasion in oesophageal carcinoma. MMP-1 mRNA was demonstrated to be mainly expressed in the stromal cells not in the tumour cells of the various carcinoma tissues including oesophageal carcinoma (Gray et al, 1993; Otani et al, 1994). Taken together, the early stage carcinoma stroma can be insufficient to respond carcinoma cells and invasion of carcinoma cells through the oesophageal wall may result from the stromal reaction against carcinoma cells.

Gelatin zymography revealed 50 kD and 40 kD bands and each band was associated with MMP-1 mRNA (Fig. 4A). The identity of 50 kD gelatinolytic band was demonstrated to be latent MMP-1 by immunoprecipitation with MMP-1 antibody (Ossowski, 1992; Kim et al, 1998) and its band was not recognized by MMP-2 antibody in the present study (Fig. 4B). This study also revealed MMP-1 to show a band at 50 kD and 40 kD, which might account for the results of gelatin zymography. Taken together, MMP-1 mRNA might represent its net function as compared with immunohistochemistry. However, MMP-1 mRNA expression inversely correlated with the prognosis, especially in advanced oesophageal carcinoma (Fig. 3, Table 2). Our results suggested MMP-1 could affect local invasion, but not systemic dissemination in oesophageal carcinoma. In breast carcinoma, cases with a 50 kD gelatinolytic band was demonstrated to show a better prognosis than the cases with no band (Remacle et al, 1996). MMP-1 might be an inverse prognostic marker even in breast carcinoma as our oesophageal carcinoma did. MMP-1 was dmeonstrated to be intensely expressed in eosinophil (Ono et al, 1997; Gray et al, 1993) as our immunohistochemistry did (Fig. 5E, F) and good carcinoma prognosis has been shown to correlate with eosinophil infiltration (Lowe et al, 1981; Pretlow et al, 1983; Fisher et al, 1989; McGinnis et al, 1989; Sier et al, 1996). Recently, other MMP expression derived from the host cell such as macrophages or neutrophils was demonstrated to reflect the host defence against carcinoma cells (Takeha et al, 1997; Migita et al, 1999). Taken together, MMP-1 expression in eosinophils may reflect this phenomenon. However, MMP-1 was also immunolocalized in carcinoma cells, which suggested the prognostic significance of MMP-1 to be unlikely to be accounted for by the host defence only.

Our results revealed that early stage oesophageal carcinomas, which were all T/N ratio < 10, did not include dead cases and showed a good prognosis as reported previously in early

 © 2001 Cancer Research Campaign

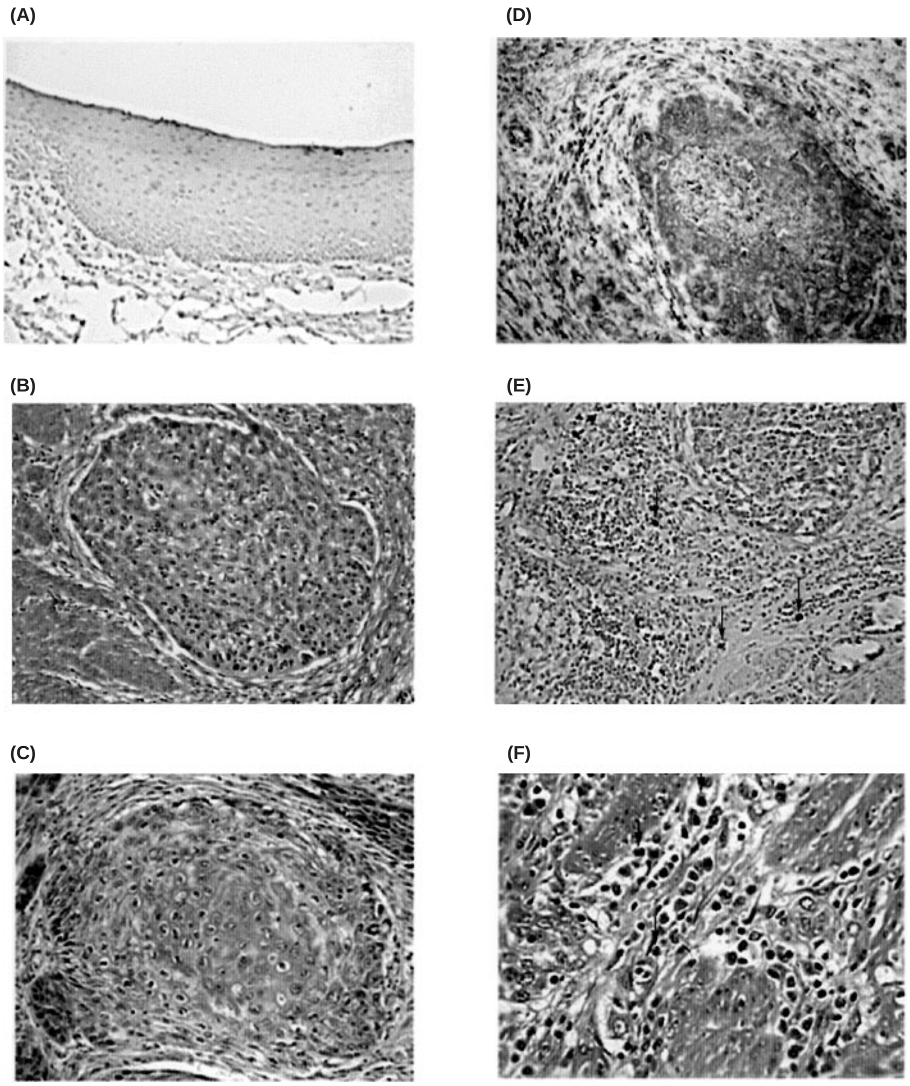

**Figure 5** Immunohistochemistry: (**A**) Normal oesophageal epithelial cells show negligible to weak immunostaining (original magnification × 100). (**B**) HE staining of carcinoma cells invading muscular layer (original magnification × 400). (**C**) The next slide of Figure 5B showed that carcinoma cells stained positive for MMP-1. (**D**) MMP-1 was also immunolocalized in stromal fibroblasts as well as carcinoma cells in 4 of 17 cases. (**E**) MMP-1 was expressed intensely in infiltrated eosinophils (arrow) around the tumours (original magnification × 200). (**F**) HE staining revealed dinucleated cells with eosinophilic cytoplasm (eosinophils) (arrow) in the next slide of Figure 5E, (original magnification × 400)

oesophageal carcinoma by Sugimachi et al (1989). On the other hand, advanced oesophageal carcinoma with a T/N ratio of <10 showed a poorer prognosis than cases with a T/N ratio of ≥10, which suggested the different significance of early stage or advanced carcinoma with a T/N ratio of <10. Baba et al (1993) demonstrated that early stage oesophageal carcinoma showed an immature form not yet infiltrated by the stromal cells. This finding indicated that early carcinoma with a MMP-1 T/N ratio of <10 included both immature stromal state and poorly responsive stromal one against carcinoma cells regarding MMP-1 mRNA expression. In contrast, advanced carcinomas with MMP-1 T/N ratio of <10 might become a mature but poorly responsive state and such cases among advanced oesophageal carcinoma seem to have a poor capacity of invasion and show a retarded spread of the primary tumour. This is concorded with the previous reports that the spread of carcinoma cells correlated with the lysis of type I collagen in carcinoma tissues (Burman and Carter, 1985; van der Stappen et al, 1990). Almost all advanced oesophageal carcinomas were discovered based on the unique symptoms such as dysphasia or hoarseness, which is thought to be determined by the primary spread. Taken together, advanced carcinoma with a T/N ratio of <10 could take a longer time for symptom to appear. On the other hand, MMP-2 and MMP-9 were expressed independently from the depth of invasion of our oesophageal carcinoma data using the same sample (data not shown) and such MMPs expression did not associate with MMP-1 expression in our analysis (data not shown) and others (Brown et al, 1990; Okada et al, 1995). These results indicated clinical oesophageal carcinoma tissues to have a metastatic potential at early stage.

In conclusion, MMP-1 is expressed exclusively in oesophageal carcinoma tissues and involved in the primary spread of carcinoma cells around the adjacent tissues at early stage. In advanced carcinoma, however, MMP-1 did not reflect the stage of systemic dissemination, and cases with T/N ratio < 10 seem to predict a

© 2001 Cancer Research Campaign 

more advanced stage regarding the systemic dissemination as compared with those with a T/N ratio of ≥10, because cases with T/N ratio < 10 could take a longer time for symptom to appear. Taken together, MMP-1 could be a novel prognostic factor independent from those previously reported in oesophageal carcinoma.

### REFERENCES

Baba K, Kuwano H, Kitamura K and Sugimachi K (1993) Carcinomatous invasion and lymphocyte infiltration in early esophageal carcinoma with special regard to the basement membrane. An immunohistochemical study. *Hepatogastroenterol* **40**: 226–231

Brooks P, Silletti S, von Schalscha T, Friendlander M and Cheresh D (1998) Disruption of angiogenesis by PEX, a noncatalytic metalloproteinase fragment with integrin binding activity. *Cell* **92**: 391–400

Brown PD, Levy AT, Margulies IMK, Liotta LA and Stetler-Stevenson WG (1990) Independent expression and cellular processing of Mr 72,000 type IV collagenase and interstitial collagenase in human tumorigenic cell lines. *Cancer Res* **50**: 6184–6191

Burman JF and Carter RL (1985) Lysis of type-I collagen by squamous carcinomas of the head and neck. *Int J Cancer* **36**: 109–116

Fisher E, Paik S, Rockette H, Jones J, Caplan R and Fisher B (1989) Prognostic significance of eosinophils and mast cells in rectal cancer. *Hum Pathol* **20**: 159–163

Gray S, Yun K, Motoori T and Kuys Y (1993) Interstitial collagenase gene expression in colonic neoplasia. *Am J Pathol* **143**: 663–671

Japanese Society for Oesophageal Disease (1976) Guidelines for the clinical and pathologic studies on carcinoma of the oesophagus. *Jpn J Surg* **6**: 69–78

Kim J, Yu W, Kavolski K and Ossowski L (1998) Requirement for specific proteases in cancer cell intravasation as revealed by a novel semiquantitative PCR-based assay. *Cell* **94**: 353–362

Liotta L, Tryggvason K, Garbisa S, Hart I, Foltz C and Shafie S (1980) Metastatic potential correlates with enzymatic degradation of basement membrane collagen. *Nature (Lond)* **284**: 67–68

Liotta L, Steeg P and Stetler-Stevenson W (1991) Cancer metastasis and angiogenesis: an imbalance of positive and negative regulation. *Cell* **64**: 327–336

Lowe D, Jorizzo J and Hutt M (1981) Tumor-associated eosinophilia: *A Review J Clin Pathol* **34**: 1343–1348

Masson R, Lefebvre O, Noel A, Fahime ME, Chenard M-P, Wendling C, Kebers F, LeMeur M, Dierich A, Foidart J-M, Basset P and Rio M-C (1998) In vivo evidence that the stromelysin-3 metalloproteinase contributes in a paracrine manner to epithelial cell malignancy. *J Cell Biol* **140**: 1535–1541

McGinnis M, EL Bradley J, Pretlow T, Ortiz-Reyes R, Bowden C, Stellato T and II TP (1989) Correlation of stromal cells by morphometric analysis with metastatic behavior of human colonic carcinoma. *Cancer Res* **49**: 5989–5993

Migita T, Sato E, Saito K, Mizoi T, Shiiba K, Matsuno S, Nagura H and Ohtani H (1999) Differing expression of MMPs-1 and -9 and urokinase receptor between diffuse-and intestinal-type gastric carcinoma. *Int J Cancer* **84**: 74–79

Mimori K, Mori M, Inoue H, Ueo H, Mafune K, Akiyoshi T and Sugimachi K (1996) Elongation factor 1 gamma mRNA expression in oesophageal carcinoma. *Gut* **38**: 66–70

Mori M, Barnard GF, Raymond J, Sttaniunas JM, Steele GD, Jr and Chen LB (1993) Prothymosin-α mRNA expression correlates with that of c-myc in human colon cancer. *Oncogene* **8**: 2821–2826

Mori M, Mimori K, Shiraishi T, Fujie T, Baba K, Kusumoto H, Haraguchi M, Ueo H and Akiyoshi T (1997) Analysis of MT1-MMP and MMP2 expression in human gastric cancers. *Int J Cancer* **74**: 316–321

Murray GI, Duncan ME, O’Neil P, Melvin WT and Fothergill JE (1996) Matrix metalloproteinase-1 is associated with poor prognosis in colorectal cancer. *Nat Med* **2**: 461–462

Murray GI, Duncan ME, Arbuckle E, Melvin WT and Fothergill JE (1998a) Matrix metalloproteinases and their inhibitors in gastric cancer. *Gut* **43**: 791–797

Murray GI, Duncan ME, O’Neil P, Mckay JA, Melvin WT and Fothergill JE (1998b) Matrix metalloproteinase-1 is associated with poor prognosis in oesophageal cancer. *J Pathol* **185**: 256–261

Okada A, Bellocq J-P, Rouyer N, Chenard M-P, Rio M-C, Chambon P and Basset P (1995) Membrane-type matrix metalloproteinase (MT-MMP) gene is expressed in stromal cells of human colon, breast, and head and neck carcinoma. *Proc Natl Acad Sci USA* **92**: 2730–2734

Ono Y, Fujii M, Kameyama K, Otani Y, Sakurai Y and Kanzai J (1997) Expression of matrix metalloproteinase-1 mRNA related to eosinophilia and interleukin-5 gene expression in head and neck tumor tissues. *Virchows Arch* **431**: 305–310

Ossowski L (1992) Invasion of connective tissue by human carcinoma cell lines: Requirement for urokinase, urokinase receptor, and interstitial collagenase. *Cancer Res* **52**: 6754–6760

Otani Y, Okazaki I, Arai M, Kameyama K, Wada N, Maruyama K, Yoshino K, Kitajima M, Hosoda Y and Tsuchiya M (1994) Gene expression of interstitial collagenase (matrix metalloproteinase 1) in gastrointestinal tract cancers. *J Gastroenterol* **29**: 391–397

Pretlow T, Keith E, Cryar K, Bartolucci A, Pitts A, Pretlow TI, Kimball P and Boohaker E (1983) Eosinophilic infiltration of human colonic carcinoma as a prognostic indicator. *Cancer Res* **43**: 2997–3000

Remacle A, Noel A, Duggan C, McRermott E, O’higgins N, Foidart J and Duffy M (1996) Assay of matrix metalloproteinases type 1, 2, 3 and 9 in breast cancer. *Br J Cancer* **77**: 925–931

Shima I, Sasaguri Y, Kusukawa J, Yamana H, Fujita H, Kakegawa T and Morimatsu M (1992) Production of matrix metalloproteinase-2 and metalloproteinase-3 related to malignant behavior of esophageal carcinoma. *A clinicopathologic study Cancer* **70**: 2747–2753

Sier C, Kubben F, Ganesh S, Heerding M, Griffioen G, Hanemaaijer R, Krieken Jv, Lamers C and Verspaget H (1996) Tissue levels matrix metalloproteinases MMP2 and MMP9 are related to the overall survival of patients with gastric carcinoma. *Br J Cancer* **74**: 413–417

Sugimachi K, Ohno S, Matsumoto H, Mori M, Matsuoka H and Kuwano H (1989) Clinicopathologic study of early stage esophageal carcinoma. *Surgery* **105**: 706–710

Takeha S, Fujiyama Y, Bamba T, Sorsa T, Nagura H and Ohtani H (1997) Stromal expression of MMP-9 and urokinase receptor is inversely associated with liver metastasis and with infiltrating growth in human colorectal cancer: a novel approach from immune/inflammatory aspect. *Jpn J Cancer Res* **88**: 72–81

van der Stappen JF, Jr, Hendriks J and Wobbes T (1990) Correlation between collagenolytic activity and grade of histological differentiation in colorectal tumors. *Int J Cancer* **45**: 1071–1078

Wilson CL, Heppner KJ, Labosky PA, Hogan BLM and Matrisian LM (1997) Intestinal tumorigenesis is suppressed in mice lacking the metalloproteinase matrilysin. *Proc Natl Acad Sci USA* **94**: 1402–1407

Woessner JE, Jr (1991) Matrix metalloproteinases and their inhibitors in connective tissue remodeling. *FASEB J* **5**: 2145–2154

Yamashita K, Mori M, Shiraishi T, Shibuta K and Sugimachi K (2000) Clinical significance of matrix metalloproteinase-7 (MMP-7) in esophageal carcinoma. *Clin Cancer Res* **6**: 1169–1174

Yoshizaki T, Sato T, Maruyama Y, Murono S, Furukawa M, Park C and Seiki M (1997) Increased expression of membrane type 1-matrix metelloproteinase in head and neck carcinoma. *Cancer* **79**: 139–144

 *© 2001 Cancer Research Campaign*
